# LncDisAP: a computation model for LncRNA-disease association prediction based on multiple biological datasets

**DOI:** 10.1186/s12859-019-3081-1

**Published:** 2019-12-02

**Authors:** Yongtian Wang, Liran Juan, Jiajie Peng, Tianyi Zang, Yadong Wang

**Affiliations:** 1 School of Computer Science and Technology, Harbin Institute of Technology, Harbin, 150001 People’s Republic of China; 2 School of Life Science and Technology, Harbin Institute of Technology, Harbin, 150001 People’s Republic of China; 3 School of Computer Science, Northwestern Polytechnical University, Xi’an, People’s Republic of China

**Keywords:** Long non-coding RNAs, Disease, lncRNA network, Random walking with restart

## Abstract

**Background:**

Over the past decades, a large number of long non-coding RNAs (lncRNAs) have been identified. Growing evidence has indicated that the mutation and dysregulation of lncRNAs play a critical role in the development of many complex human diseases. Consequently, identifying potential disease-related lncRNAs is an effective means to improve the quality of disease diagnostics and treatment, which is the motivation of this work. Here, we propose a computational model (LncDisAP) for potential disease-related lncRNA identification based on multiple biological datasets. First, the associations between lncRNA and different data sources are collected from different databases. With these data sources as dimensions, we calculate the functional associations between lncRNAs by the recommendation strategy of collaborative filtering. Subsequently, a disease-associated lncRNA functional network is built with functional similarities between lncRNAs as the weight. Ultimately, potential disease-related lncRNAs can be identified based on ranked scores derived by random walking with restart (RWR). Then, training sets and testing sets are extracted from two different versions of a disease-lncRNA dataset to assess the performance of LncDisAP on 54 diseases.

**Results:**

A lncRNA functional network is built based on the proposed computational model, and it contains 66,060 associations among 364 lncRNAs associated with 182 diseases in total. We extract 218 known disease-lncRNA pairs associated with 54 diseases to assess the network. As a result, the average AUC (area under the receiver operating characteristic curve) of LncDisAP is 78.08%.

**Conclusion:**

In this article, a computational model integrating multiple lncRNA-related biological datasets is proposed for identifying potential disease-related lncRNAs. The result shows that LncDisAP is successful in predicting novel disease-related lncRNA signatures. In addition, with several common cancers taken as case studies, we found some unknown lncRNAs that could be associated with these diseases through our network. These results suggest that this method can be helpful in improving the quality for disease diagnostics and treatment.

## Background

Long non-coding RNAs (lncRNAs), which compose the largest portion of the mammalian non-coding transcriptome [1], are emerging as important regulators of tissue physiology and disease processes [2]. lncRNAs are expressed in a more tissue-specific fashion than mRNA genes [3] and are highly specific to cell type‚ organs‚ and species [4]. A large amount of lncRNAs have been demonstrated to have a close relationship with many complex human diseases [5–8]. Therefore, an increasing recognition of the roles of lncRNAs in human disease has created new diagnostic and therapeutic opportunities [9]. The identification of potential lncRNAs related to complex diseases is a hot topic in medicine.

LncRNAs are the key to explaining disease mechanisms. As analysing lncRNAs is very appealing to researchers, many researchers have devoted their work to lncRNAs for exploring complex human diseases at the molecular level. For example, BCYRN1 has been demonstrated to induce the proliferation and migration of non-small cell lung cancer (NSCLC) cells and play an important role in NSCLC progression [10]. LncRNA SNHG1 regulates NOB1 expression by sponging miR-326 and promotes tumourigenesis in osteosarcoma [11]. Ye et al. found that LINC00460 promotes the progression of lung adenocarcinoma by competitively binding miR-302c-5p and regulating the FOXA1 signalling pathway [12]. F. Aksoy et al. postulated that the overexpression of lncRNA DANCR may be associated with poor outcomes in upper rectal cancer [13]. LncRNA HOTAIR plays a role as an oncogenic molecule in different cancers, including breast, gastric, colorectal and cervical cancer cells [14]. Similarly, lncRNA MALAT1 is considered a potential biomarker for the diagnosis and prediction of cancers and may also serve as a therapeutic target for the treatment of specific tumours [15]. In 2018, Chen C et al. deduced that the expression of lncRNA ZEB1-AS1 might be used as a promising prognostic biomarker for cancer [16]. The above studies show that lncRNAs have been recently regarded as possible biomarkers for disease.

Although a large number of lncRNAs have been recorded in public databases, such as GENCODE [17], NONCODE [18], LNCipedia [19], only a few lncRNAs have been characterized functionally [20]. Several methods have been developed to predict potential lncRNA-disease associations [21, 22]. However, they take into account only disease semantic similarity and ignore disease functional similarity. Improved knowledge has suggested that exploring both the semantic and functional associations of diseases, which are two types of significant associations, are beneficial in measuring disease similarity because not all associations between diseases are represented by the disease ontology, and many of them are reflected through the functional associations among disease-related genes [23]. Moreover, the lack of unified identifications for lncRNAs leads to an underutilization of information from different public lncRNA databases when lncRNA functional annotations are approached. Therefore, we aimed to identify more lncRNAs by efficiently analysing the lncRNA and disease data. First, we extracted and utilized functional information related to lncRNAs, including disease similarity, protein-protein interactions and lncRNA-mRNA associations. Subsequently, we established functional associations between lncRNAs and built a disease-related lncRNA network. Potential disease-related lncRNA signatures were predicted by a random walking with restart (RWR).

## Materials and methods

### Workflow

**Fig. 1:**
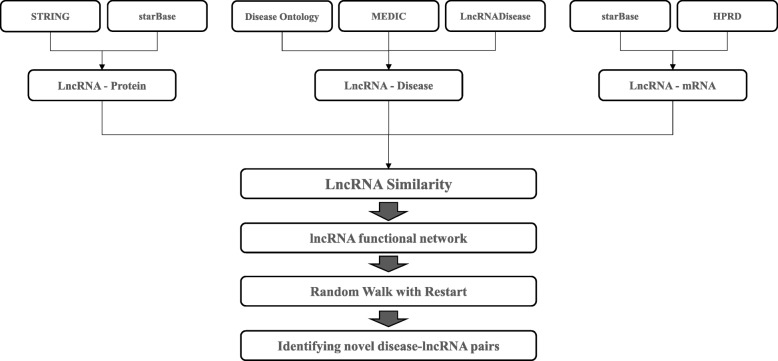
The workflow of LncDisAP for identifying potential disease-related lncRNAs

The workflow of LncDisAP is shown in Fig. 1. First, mappings between lncRNAs and lncRNA-related datasets are established, and these datasets are extracted from multiple biological datasets. Mappings between lncRNA and protein are provided by the Search Tool for the Retrieval of Interacting Genes/Proteins (STRING) [24] and starBase v2.0 [25] databases, while those between lncRNA and disease are from the Human Disease Ontology (DO) [26], MEDIC [27] and LncRNADisease [28] databases. The mappings of lncRNA-mRNA are from starBase v2.0 [25] and the Human Protein Reference Database (HPRD) [29]. Subsequently, different similarity measures can be defined considering that different data sources have different data characteristics. Given the associations between lncRNA and mRNA, the number of lncRNA-related mRNAs can be taken as a statistical indicator to calculate lncRNA similarity. In view of disease functional similarity and protein functional similarity, associations of lncRNA-disease and lncRNA-protein are used to make a multi-dimensional vector model for each lncRNA. Finally, a disease-related lncRNA functional network is built based on lncRNA functional similarity. We employ RWR in this network to calculate the ranking of candidate lncRNAs, which are related to certain diseases. Thus, the potential relationships between diseases and lncRNAs can be identified.

## Data source

### Disease database

DO [26] database is focused on representing a common and rare disease concept, which aims to provide an open source ontology for the integration of biomedical data associated with human disease. Each node in DO represents one disease term. All of these nodes are organized in a directed acyclic graph (DAG) with an ‘IS_A’ relationship. MEDIC [27], as a part of the Comparative Toxicogenomics Database (CTD) [30], integrates Online Mendelian Inheritance in Man (OMIM) terms, synonyms and identifiers with MeSH [31] terms, synonyms, definitions, identifiers and hierarchical relationships. It is composed of 9700 unique diseases described by more than 67,000 terms. In this study, we map lncRNA-related diseases to DO, utilizing terms and synonyms from DO and MEDIC.

### LncRNA database

RNAcentral [32] is a database of non-coding RNA (ncRNA) sequences that aggregates data from specialized ncRNA resources. It assigns unique identifiers to every distinct RNA sequence. Because there is no uniform identity number in the different lncRNA databases, we use identifiers from RNAcentral as unified labels of lncRNAs to ensure the smooth progress of this work.

### Human lncRNA-disease association data

LncRNADisease [28] is a database that curated the experimentally supported lncRNA-disease association data. Presently, there are three versions available. The 2017 version of the LncRNADisease database integrated 2947 lncRNA-disease entries, including 888 lncRNAs and 328 diseases, while the 2015 version covered 1102 lncRNA-disease entries, including 373 lncRNAs and 252 diseases. The newest version [33] was released in 2018, containing 5714 lncRNAs and 423 diseases. Here, we extract associations between lncRNAs and diseases from this database and use the differences between its versions to validate the reliability of LncDisAP.

### Human protein-protein interaction data

STRING [24] is a database of known and predicted protein-protein interactions. These interactions in STRING include direct (physical) interactions, as well as indirect (functional) interactions, which stem from computational prediction, knowledge transfer between organisms, and interactions aggregated from other databases. The STRING database currently covers 9,643,763 proteins from 2031 organisms. Here, protein-protein interactions from STRING are involved in the lncRNA similarity computation.

### Human lncRNA interaction data

starBase v2.0 [25] systematically identified the RNA-RNA and protein-RNA interaction networks from 108 CLIP-Seq data sets generated by 37 independent studies, which provided 423,966 miRNA-mRNA, 10,212 miRNA-lncRNA and 17,609 protein-lncRNA experimentally confirmed interactions based on large scale CLIP-Seq data. The HPRD [29] represents an mRNA-mRNA interaction network for humans. All the information in HPRD has been manually extracted from the literature by expert biologists. Currently, HPRD covers 39,240 mRNA-mRNA interactions with 9465 mRNA.

## LncRNA functional similarity calculation

### Data pre-processing

The differences in different data sets bring some difficulties to the integration of lncRNA data. Two problems must be solved before constructing the lncRNA functional association network. One is the mapping of disease terms. MEDIC and DO are both comprehensive disease corpuses and contain abundant disease terms, so we can annotate DO entries with the vocabulary from MEDIC and create a combined vocabulary of disease terms. Referring to this vocabulary, we build mappings between the DO terms and the disease terms of LncRNADisease. The other problem that must be addressed is the unification of lncRNA identifications. As mentioned above, the lncRNA naming rules of different lncRNA databases are different. Therefore, we employ the RNAcentral id as the unified identification system of lncRNAs considering that the RNAcentral database provides mapping data among various public lncRNA databases.

### LncRNA-related disease similarity

The functional similarities between different diseases can be calculated. Therefore, in view of associations between lncRNAs and diseases, we can make a multi-dimensional vector model for each lncRNA with diseases as dimensions. The functional similarities between these lncRNA-related diseases can be taken as inputs to further calculate relevance scores for lncRNAs. Here, we employ FNSemSim [34] to calculate disease functional similarity. This method, which we previously developed, has good performance for calculating similarities between diseases. In this method, we first calculate disease functional similarity utilizing associations between diseases and genes. The functional similarity between disease *d*_*a*_ and *d*_*b*_ is defined as follows:

1 $$ FNSim\left({G}_a,{G}_b\right)=\frac{\sum \limits_{1\le i\le num\left({G}_a\right)}{R}_{G_b}\left({g}_{ai}\right)+\sum \limits_{1\le j\le num\left({G}_b\right)}{R}_{G_a}\left({g}_{bj}\right)}{num\left({G}_a\right)+ num\left({G}_b\right)} $$

$$ {g}_{ai}\in {G}_a,{g}_{bj}\in {G}_b $$

where the gene sets *G*_*a*_ = {*g*_*a*1_, *g*_*a*2_, …} and *G*_*b*_ = {*g*_*b*1_, *g*_*b*2_, …} are related to disease *d*_*a*_ and *d*_*b*_, respectively; *num*(*G*) represents the numbers of genes related to one disease; and *R*_*G*_(*g*) represents the degree of association between a gene *g* and a gene set *G* (see details in [34]). Considering that sematic associations exist in DO, FNSemSim could be defined as follows:

2 $$ FNSemSim\left({d}_a,{d}_b\right)= FNSim\left({G}_a,{G}_b\right)\ast \frac{\left|{G}_a\right|\left|{G}_b\right|}{{\left|{G}_{MICA}\right|}^2} $$

where |*G*| represents the size of a gene set *G*. *G*_*MICA*_ represents the genes related to the most informative common ancestor of disease *d*_*a*_ and *d*_*b*_. Finally, by min-max normalization, we normalize similarities between pair-wised diseases.

### Vector model construction for lncRNAs

**Fig. 2:**
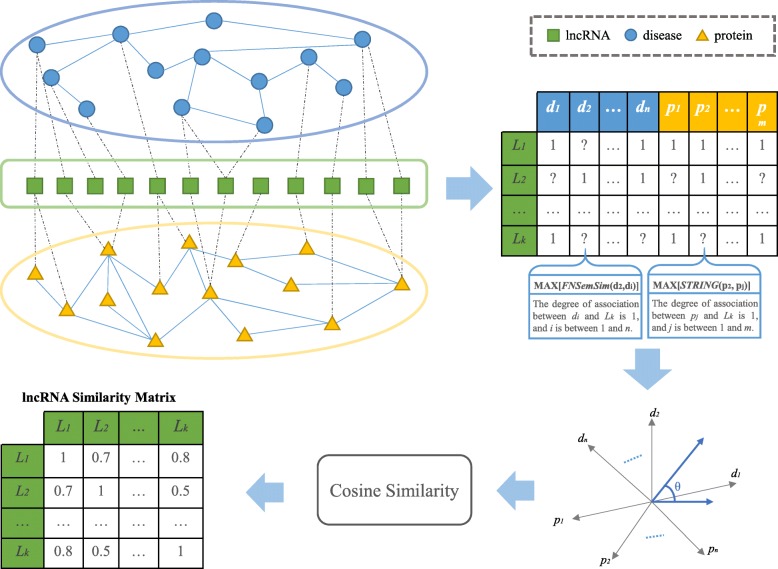
The workflow of calculating lncRNA similarity based on the recommendation strategy

STRING provides human protein-protein interactions, and in the above section, the functional similarities between lncRNA-related diseases have been calculated. Therefore, we can obtain the relational degrees between one lncRNA and a certain disease or protein based on the similarities of lncRNA-related diseases or proteins. Then, these degrees can be used to make a multi-dimensional vector for each lncRNA. Hence, we can calculate lncRNA functional similarity by cosine similarity in a multi-dimensional space, which is defined by lncRNA-related diseases and proteins. The workflow of calculating lncRNA functional similarity based on the recommendation strategy of collaborative filtering is shown in Fig. 2.

In this multi-dimensional space, neither all diseases nor all proteins are directly related to one lncRNA. To predict the score of a disease that is not directly related to one lncRNA, we define *L* as the set of lncRNAs, *D* as the set of lncRNA-related diseases and *P* as the set of lncRNA-related proteins. *DR*_*l*_ is defined as the set of diseases directly related to lncRNA *l*. The predicted association score between disease *d* and lncRNA *l* is defined as follows:

3 $$ AS\left(d,l\right)=\left\{\begin{array}{c}\operatorname{MAX}\left( FNSemSim\left({d}_i,d\right)\right)\\ {}1\end{array}\begin{array}{cc}& \\ {}& \end{array}\begin{array}{l}{d}_i\in D{R}_l\kern0.5em and\kern0.5em d\notin D{R}_l\\ {}d\in D{R}_l\end{array}\right) $$

where *l* ∈ *L*, *d* ∈ *D*, *DR*_*l*_ ⊆ *D* and 1 ≤ *i* ≤ |*DR*_l_|; here, |*DR*_*l*_| represents the number of diseases in the set of *DR*_*l*_. Similarly, for lncRNA-related proteins, *PR*_*l*_ is defined as the set of proteins directly related to lncRNA *l*. The predicted association score between protein *p* and lncRNA *l* is defined as follows:

4 $$ AS\left(p,l\right)=\left\{\begin{array}{c}\operatorname{MAX}\left( SPscore\left({p}_i,p\right)\right)\\ {}1\end{array}\begin{array}{cc}& \\ {}& \end{array}\begin{array}{l}{p}_i\in P{R}_l\kern0.5em and\kern0.5em p\notin P{R}_l\\ {}p\in P{R}_l\end{array}\right) $$

where *l* ∈ *L*, *p* ∈ *P*, *PR*_*l*_ ⊆ *P* and 1 ≤ *i* ≤ |*PR*_*l*_|; here, |*PR*_*l*_| represents the number of proteins in the set of *PR*_*l*_ and *SPscore (p*_*i*_*,p)* represents the relevance score between protein *p* and *p*_*i*_ from STRING.

Subsequently, we define a vector of each lncRNA with |*D*| + |*P*| dimensions. |*D*| and |*P*| represent the size of the disease set *D* and the protein set *P*, respectively. For each lncRNA, we can define its vector $\overrightarrow{\mathrm{l}}$ as follows:

5 $$ {\displaystyle \begin{array}{c}\overrightarrow{\mathrm{l}}=\left( AS\left({d}_1,l\right),\kern0.5em \cdots \cdots, \kern0.5em AS\left({d}_k,l\right), AS\left({p}_1,l\right),\cdots \cdots, AS\left({p}_j,l\right)\right)\\ {}l\in L,\kern0.5em 1\le k\le \mid D\mid, 1\le j\le \mid P\mid \end{array}} $$

where $\overrightarrow{\mathrm{l}}$ represents the vector of lncRNA *l* in this multi-dimensional space. *AS*(*d*_*k*_, *l*) and *AS*(*p*_*j*_, *l*) are the scores of disease *d*_*k*_ and protein *p*_*j*_, respectively, for lncRNA *l*. Now, we can obtain |*L*| vectors of lncRNAs.

### LncRNA functional similarity

In this multi-dimensional space, each lncRNA can be depicted by a multi-dimensional vector. Therefore, we can measure the similarity between any two vectors of lncRNAs based on cosine similarity. The similarity between lncRNA *l*_*1*_ and lncRNA *l*_*2*_ is defined as follows:

6 $$ CR\left({l}_1,{l}_2\right)=\frac{\sum_1^{\mathrm{n}}\left({AS}_{1,i}\times {AS}_{2,\mathrm{i}}\right)}{\sqrt{\sum_1^{\mathrm{n}}{AS}_{1,i}^2}\times \sqrt{\sum_1^{\mathrm{n}}{AS}_{2,i}^2}} $$

where *AS*_*k,i*_ represents the association score in the *i*-th dimension of the vector ${\overrightarrow{l}}_{\mathrm{k}}$ for lncRNA *l*_*k*_. The range of *CR*(*l*_*1*_, *l*_*2*_) is 0 to 1 because these values of *AS*_*k,i*_ are positive numbers.

In addition, mRNA can also be seen as a factor to calculate lncRNA functional similarity because of the existing links between lncRNAs and mRNAs. In view of the relationships between mRNAs, we can extract links between them from HPRD denoted as *mRNALinkSet*. First, the relevance between an mRNA *k* and an mRNA set *M* is defined as follows:

7 $$ R\left(k,M\right)=\Big\{{\displaystyle \begin{array}{c}1\\ {} links\left(k,M\right)/\mid M\mid \end{array}}{\displaystyle \begin{array}{cc}& \\ {}& \end{array}}{\displaystyle \begin{array}{l}k\in M\\ {}k\notin M\end{array}}\operatorname{} $$

where *links(k,M)* represents the number of links between mRNA *k* and members in the mRNA set *M*, and these links have to be included in *mRNALinkSet*. Let a pair of mRNA sets *M*_1_ = {*m*_11_, *m*_12_, …} and *M*_2_ = {*m*_21_, *m*_22_, …} be related to lncRNA *l*_*1*_ and *l*_*2*_, respectively. The similarity between lncRNA *l*_*1*_ and *l*_*2*_ based on mRNA is defined as follows:

8 $$ MR\left({l}_1,{l}_2\right)=\frac{\sum \limits_{1\le i\le \mid {M}_1\mid }R\left({m}_{2i},{M}_1\right)+\sum \limits_{1\le j\le \mid {M}_2\mid }R\left({m}_{1j},{M}_2\right)}{\mid {M}_1\mid +\mid {M}_2\mid } $$

where |*M*_1_| and |*M*_2_| represent the numbers of mRNAs related to lncRNA *l*_*1*_ and *l*_*2*_, respectively. Finally, we complete the calculation of lncRNA similarities based on different lncRNA-related knowledge.

## Identifying novel candidate disease-related lncRNAs

We can take lncRNA similarities as weight to construct a lncRNA functional association network. In this network, the weight between lncRNA *l*_*1*_ and *l*_*2*_ is defined as follows:

9 $$ LncFunNet\left({l}_1,{l}_2\right)=1\hbox{-} \left(1\hbox{-} CR\left({l}_1,{l}_2\right)\right)\left(1\hbox{-} MR\left({l}_1,{l}_2\right)\right) $$

where the range of *LncFunNet*(*l*_*1*_*,l*_*2*_) is 0 to 1, as in *CR*(*l*_*1*_*,l*_*2*_) and *MR*(*l*_*1*_*,l*_*2*_). Utilizing this lncRNA network, we can identify novel candidate disease-related lncRNAs.

To identify novel candidate disease-related lncRNAs, we employ RWR to fully exploit the global functional associations between lncRNAs in this network. RWR, as a global optimization method, can reveal more information between one lncRNA and all the others in the network. The random walker in the network starts from the root node and moves to adjacent nodes with the probabilities from that node to the others. After enough iterations, the probabilities from the root node to all the other nodes will become stable, which can be used as scores for predicting novel disease-related lncRNAs (see [35] for RWR details). Finally, rankings for each lncRNA in this network can be listed by RWR.

## Results

### LncRNAs and diseases

We obtained 3,801,586 associations among 4703 disease terms from DO based on disease similarity calculations. Meanwhile, we found 1083 relationships between 184 diseases and 374 lncRNAs by mapping DO terms to the diseases in LncRNADisease (released in July 2017). There were 5,600,133 relationships between 13,716 mRNA and 1034 lncRNAs extracted from starBase v2.0. We found 15,622 associations between 33 proteins and 2750 lncRNAs from starBase v2.0 and STRING.

We calculated similarity among 374 lncRNAs and removed lncRNA pairs that had a similarity of 0. Finally, we built a lncRNA functional network, which contains 66,060 associations among 364 lncRNAs associated with 182 diseases.

### Performance

To assess the performance of the lncRNA functional network, we compared two different versions of LncRNADisease and extracted 218 known disease-lncRNA pairs associated with 54 diseases from the newer version of LncRNADisease (released in June 2018). The detailed statistics for evaluating disease-related lncRNA networks are given in Additional file 1. For each of these 54 diseases, all of the tested lncRNAs, which exist in the two different versions like other lncRNAs involved in the performance evaluation, have associations with their respective disease only in the newer version of LncRNADisease. Take cholangiocarcinoma (DOID:4947) as an example. There was only one lncRNA associated with cholangiocarcinoma in LncRNADisease (released in July 2017), while five new lncRNAs were included in the newer version of LncRNADisease. We tried to validate the performance of the lncRNA functional network for predicting associations between the five lncRNAs and cholangiocarcinoma. The information of these lncRNAs associated with cholangiocarcinoma is shown in Table 1.

**Table 1:** Information on lncRNAs associated with cholangiocarcinoma

LncRNA ID	LncRNA Name
URS0000524E5C	PANDAR
URS000075E0F9	AFAP1-AS1
URS000075ADFF	CCAT1
URS000010576B	CCAT2
URS0000812019	SPRY4-IT1

**Fig. 3:**
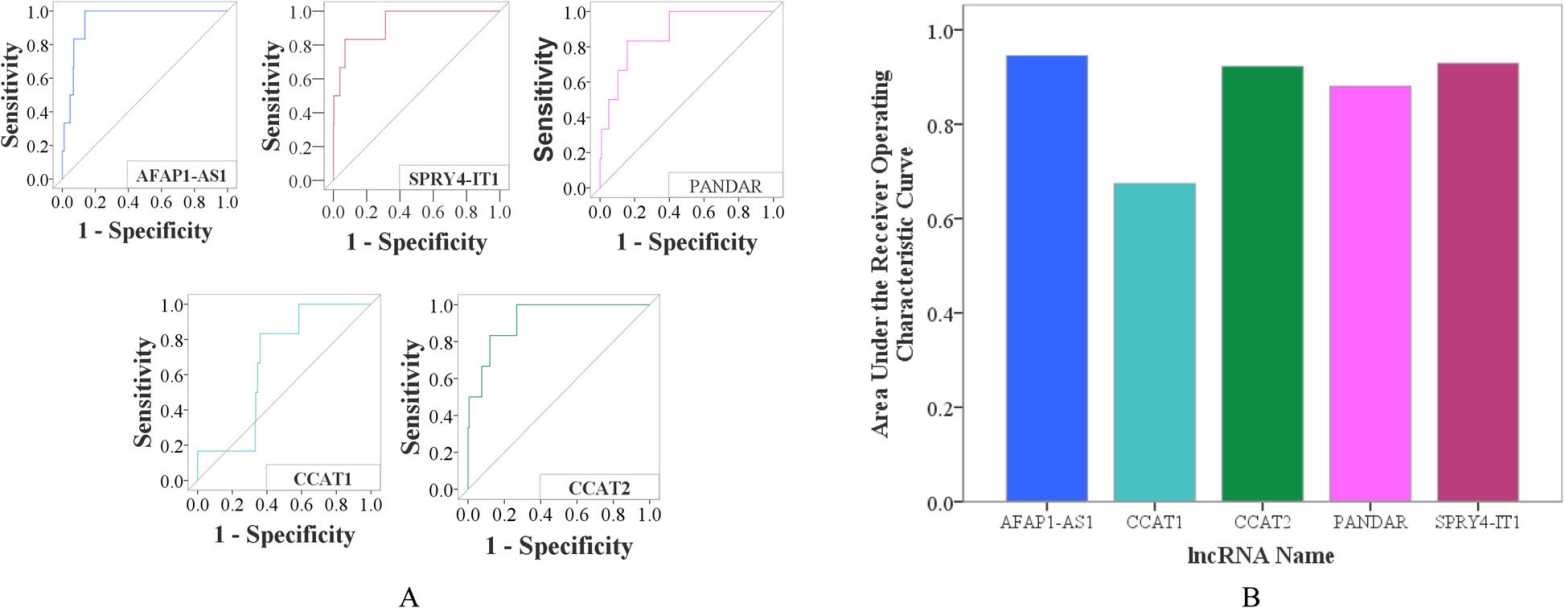
The performance in predicting candidate lncRNAs associated with cholangiocarcinoma. **a**. ROC curves of these five lncRNAs based on the test set from the 2018 version, including AFAP1-AS1, SPRY4-IT1, PANDAR, CCAT1 and CCAT2. **b**. AUC of these five lncRNAs based on the test set from the 2018 version

**Fig. 4:**
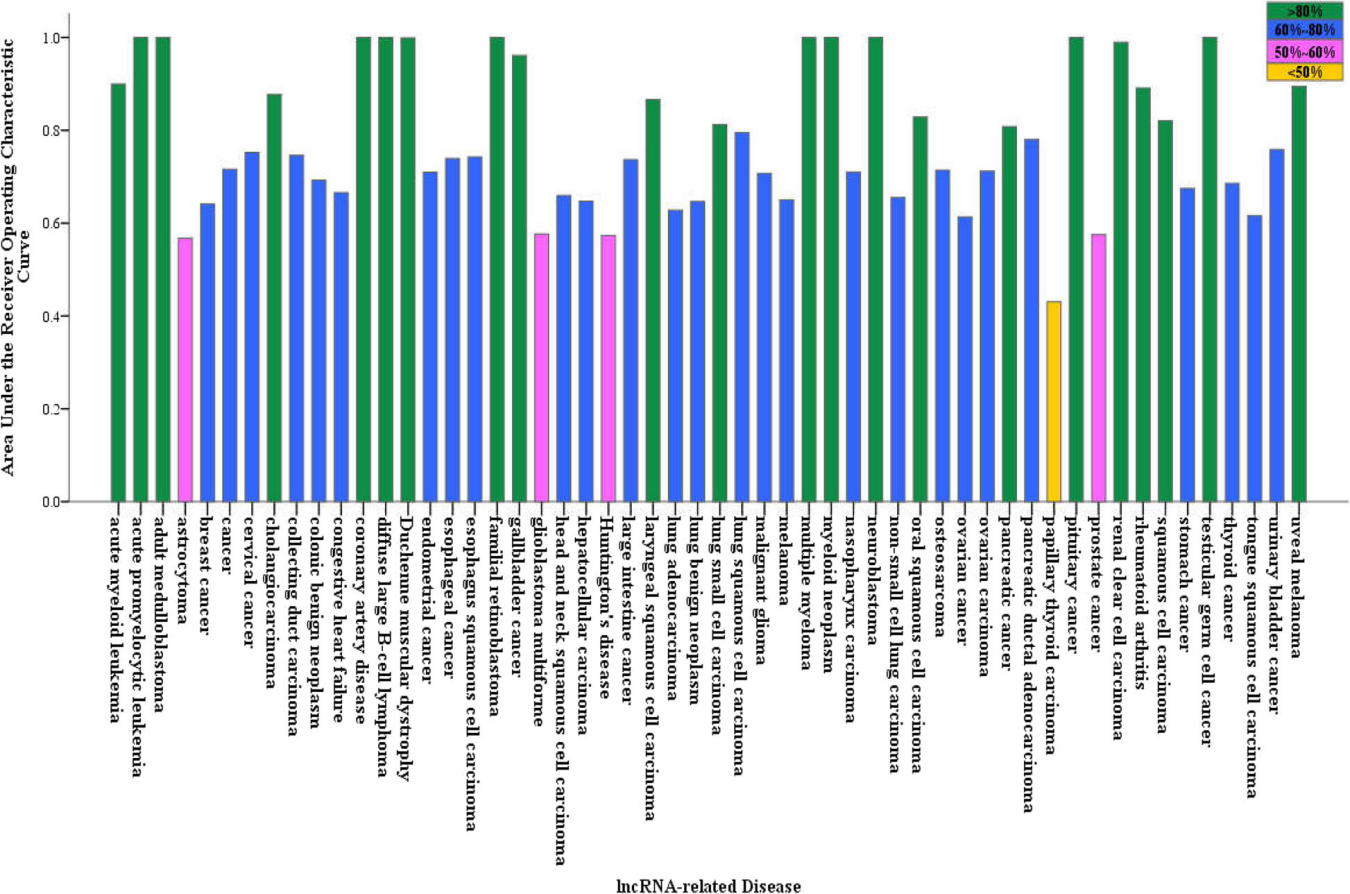
Average AUC of 54 diseases based on the test set of the 2018 version. 22 diseases have average AUC values greater than 80%, while 27 diseases have average AUC values between 60 and 80%

As a result, the disease-related lncRNA functional network has a good performance in predicting disease-lncRNA pairs for the 54 diseases with an average AUC value of 78.08%. The performance in predicting lncRNAs associated with cholangiocarcinoma is shown in Fig. 3. Meanwhile, we found that LncDisAP has outstanding performance on some diseases. For example, gallbladder cancer (DOID:3121) had an AUC of 96.13% in this lncRNA functional network. There were 22 diseases in these 54 diseases whose AUC were more than 80%, as shown in Fig. 4. However, papillary thyroid carcinoma (DOID:3969) had a small AUC value of 43.02%. We found that LncRNADisease (released in June 2018) added a new lncRNA associated with papillary thyroid carcinoma but removed 6 lncRNAs related to this disease in comparison with the version released in July 2017. This may have contributed to a poor performance for finding lncRNAs associated with papillary thyroid carcinoma owing to the effect of noise in the data source. Even so, the performance of the lncRNA functional network based on our computational model is remarkable in predicting candidate disease-related lncRNAs. The average AUC values for these 54 diseases are shown in Fig. 4.

### Case study

**Fig. 5:**
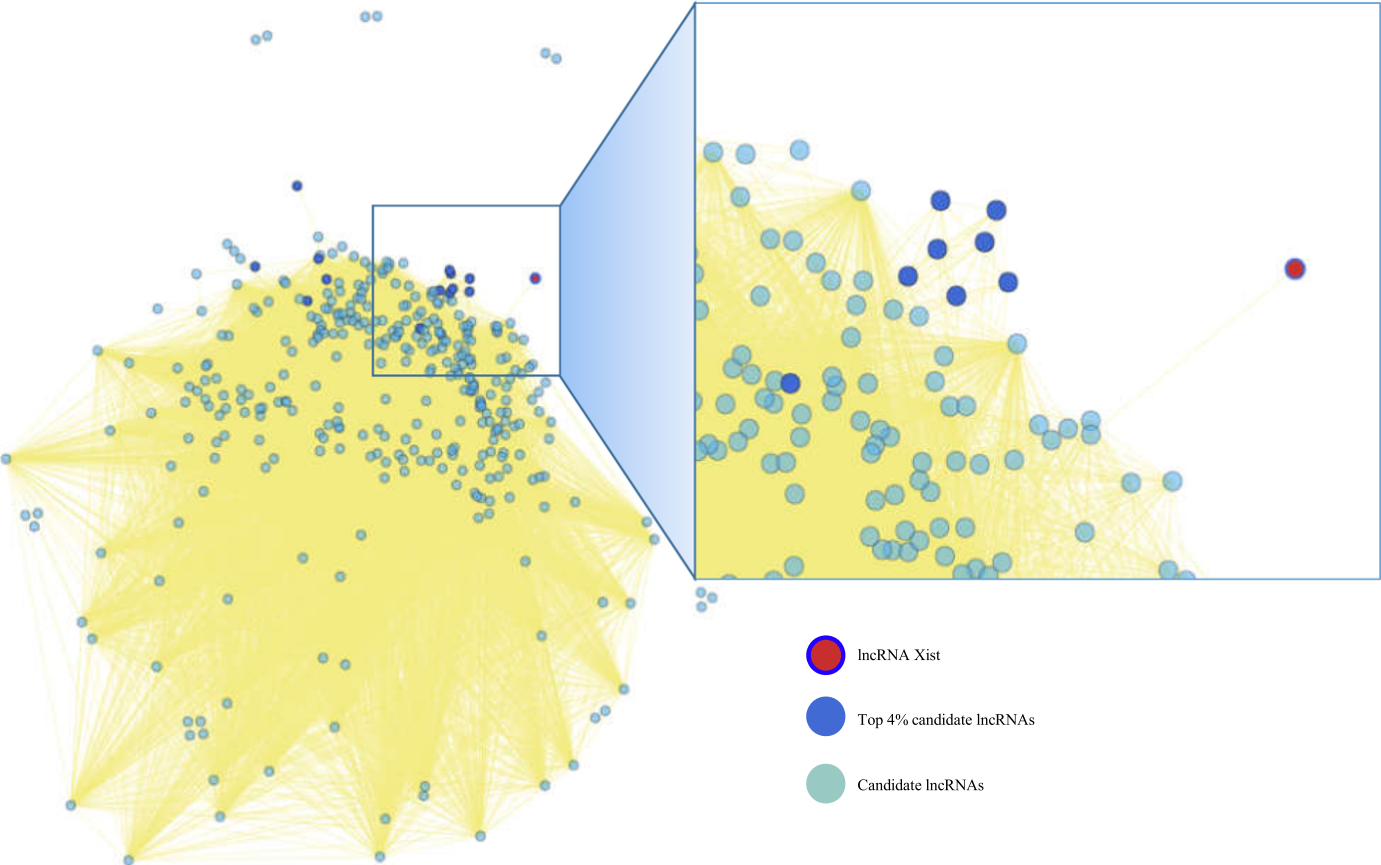
The lncRNA functional network based on the data source from the 2017 version. The threshold of associations between lncRNAs is set as 0.4 because there are a large number of associations with low scores. The top 4% ranked candidate lncRNAs for cervical cancer are shown. The green, blue and red nodes represent candidate lncRNAs, top 4% candidate lncRNAs and lncRNA Xist in top 4%, respectively

Many studies have indicated that lncRNAs play critical roles in the development of various cancers [36]. To further evaluate the performance of our computational model in predicting potential disease-related lncRNAs, we used acute myeloid leukaemia, breast cancer, cholangiocarcinoma and cervical cancer as case studies. First, we built a lncRNA functional network based on the data source from LncRNADisease (released in 2017), and the unknown lncRNA-disease associations of each disease were ranked by RWR. We found that lncRNA H19 had a high score of 0.84 for acute myeloid leukaemia, which was ranked in the top 8% and not included in the latest version of LncRNADisease. Zhang et al. [37] and Zhao et al. [38] showed that lncRNA H19 is associated with acute myeloid leukaemia. For breast cancer, lncRNA Pvt1, which was ranked in top 5%, was validated to regulate triple-negative breast cancer through KLF5/beta-catenin signalling [39]. LncRNA AFAP1-AS1 and wrap53 were both ranked in top 5% for cholangiocarcinoma and had been studied to understand cholangiocarcinoma [40–42]. Furthermore, lncRNA XIST had a top ranking of 4% for cervical cancer, as shown in Fig. 5. Zhu et al. [43] explored the specific mechanism and biological function of lncRNA XIST in cervical cancer, and their experiments indicated that lncRNA XIST accelerates the progression of cervical cancer via upregulating Fus through competitively binding with miR-200a.

## Discussion

### The impact of data sources and test sets

**Fig. 6:**
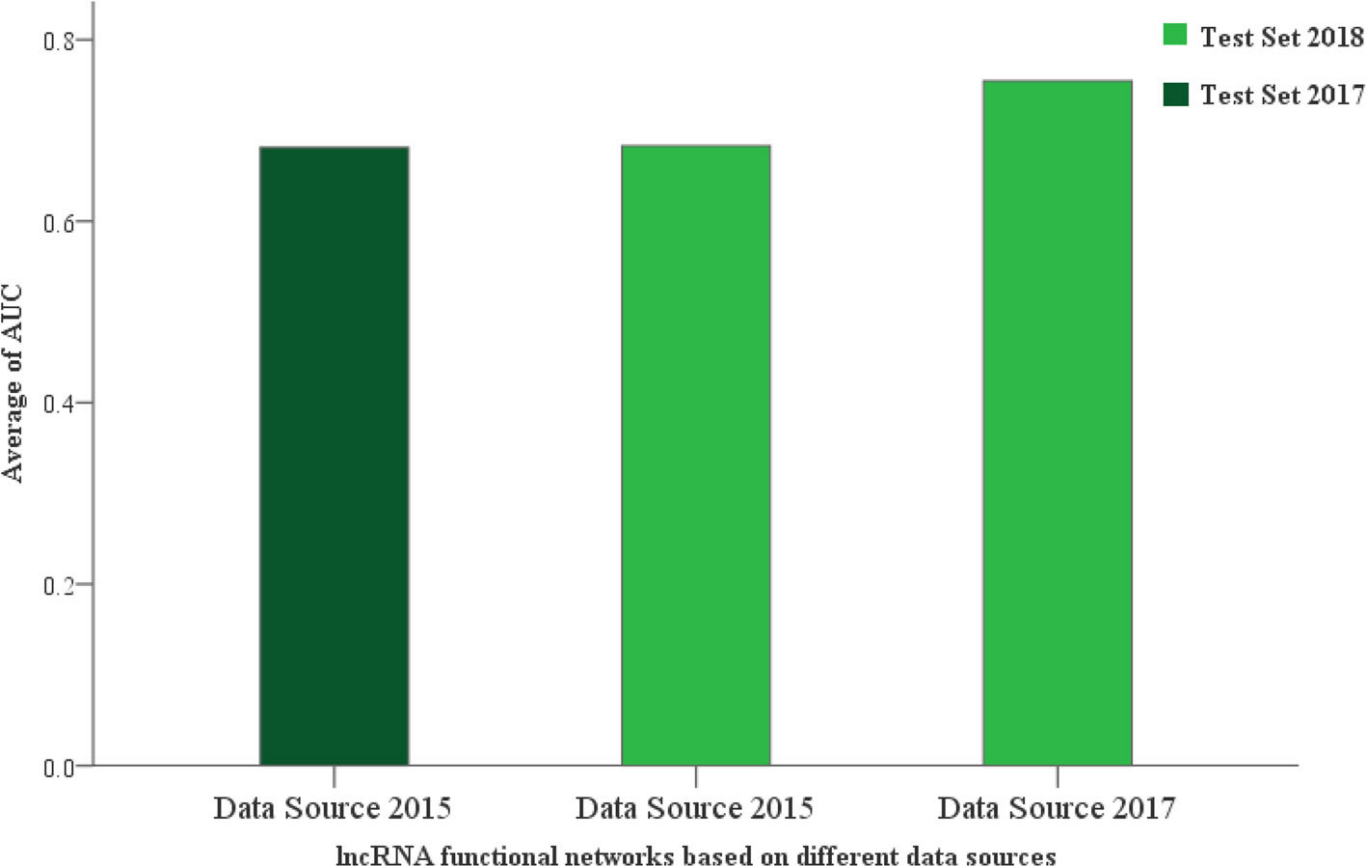
Average AUC of different lncRNA functional networks with different test sets

The relationship between diseases and lncRNAs in the data source was extracted from LncRNADisease released in July 2017. Hence, we evaluated the impact of different data sources and different test sets on the performance of predicting disease-lncRNA pairs in the disease-related lncRNA functional network. First, with the LncRNADisease data set released in June 2018 as the test set, we compared the two lncRNA functional networks that were built based on data sources from the 2015 and 2017 versions. After the abovementioned validation strategy was carried out, the lncRNA functional network based on the data source from LncRNADisease released in 2015 had an AUC value of 72.6%, while the AUC of the network based on the 2017 version reached 78.08%. Simultaneously, we assessed the performance of the lncRNA functional network based on the data source of the 2015 version with a test set extracted from the 2017 version, whose AUC reached 72.8%, as shown in Fig. 6. The test result of the lncRNA functional network based on the data source from LncRNADisease released in 2015 is given in Table 2. It can be seen that there is not much difference between the two test sets, which may be the reason why the AUCs of the network based on the two test sets do not have much difference. This suggests that the performance of predicting potential disease-lncRNA pairs in the disease-related lncRNA functional network can be noticeably impacted by different data sources and different test sets.

**Table 2:** The test result based on different versions of the data source

Disease Name	Disease Ontology	2017 Version	2018 Version
lung benign neoplasm	DOID:3683	0.6744	0.7479
stomach cancer	DOID:10534	0.7789	0.785
nasopharynx carcinoma	DOID:9261	0.6279	0.6607
lung adenocarcinoma	DOID:3910	0.6865	0.7433
squamous cell carcinoma	DOID:1749	–	0.7354
malignant glioma	DOID:3070	0.8566	0.7659
ovarian cancer	DOID:2394	0.7364	0.5976
cancer	DOID:162	–	0.7192
breast cancer	DOID:1612	0.7534	0.7847
melanoma	DOID:1909	–	0.6667
large intestine cancer	DOID:5672	0.7859	0.7275
non-small cell lung carcinoma	DOID:3908	0.6538	0.7782
**Average value of AUC**		0.7282	0.726

### LncRNA expression similarity

The introduction of lncRNA expression similarity has been considered before. However, the results are not ideal. We obtained lncRNA expression profiles from NONCODE [18]. This database is an integrated knowledge database that provides expressed profiles from human lncRNAs. Spearman’s rank correlation coefficient is employed to calculate associations between lncRNA *l*_*1*_ and *l*_*2*_, denoted as *ER(l*_*1*_*, l*_*2*_*)*. The similarity between lncRNA *l*_*1*_ and *l*_*2*_ is defined as follows:

10 $$ LncFunNet\left({l}_1,{l}_2\right)= 1\hbox{-} \left( 1\hbox{-} CR\left({l}_1,{l}_2\right)\right)\left( 1\hbox{-} MR\left({l}_1,{l}_2\right)\right)\left( 1- ER\left({l}_1,{l}_2\right)\right) $$

**Fig. 7:**
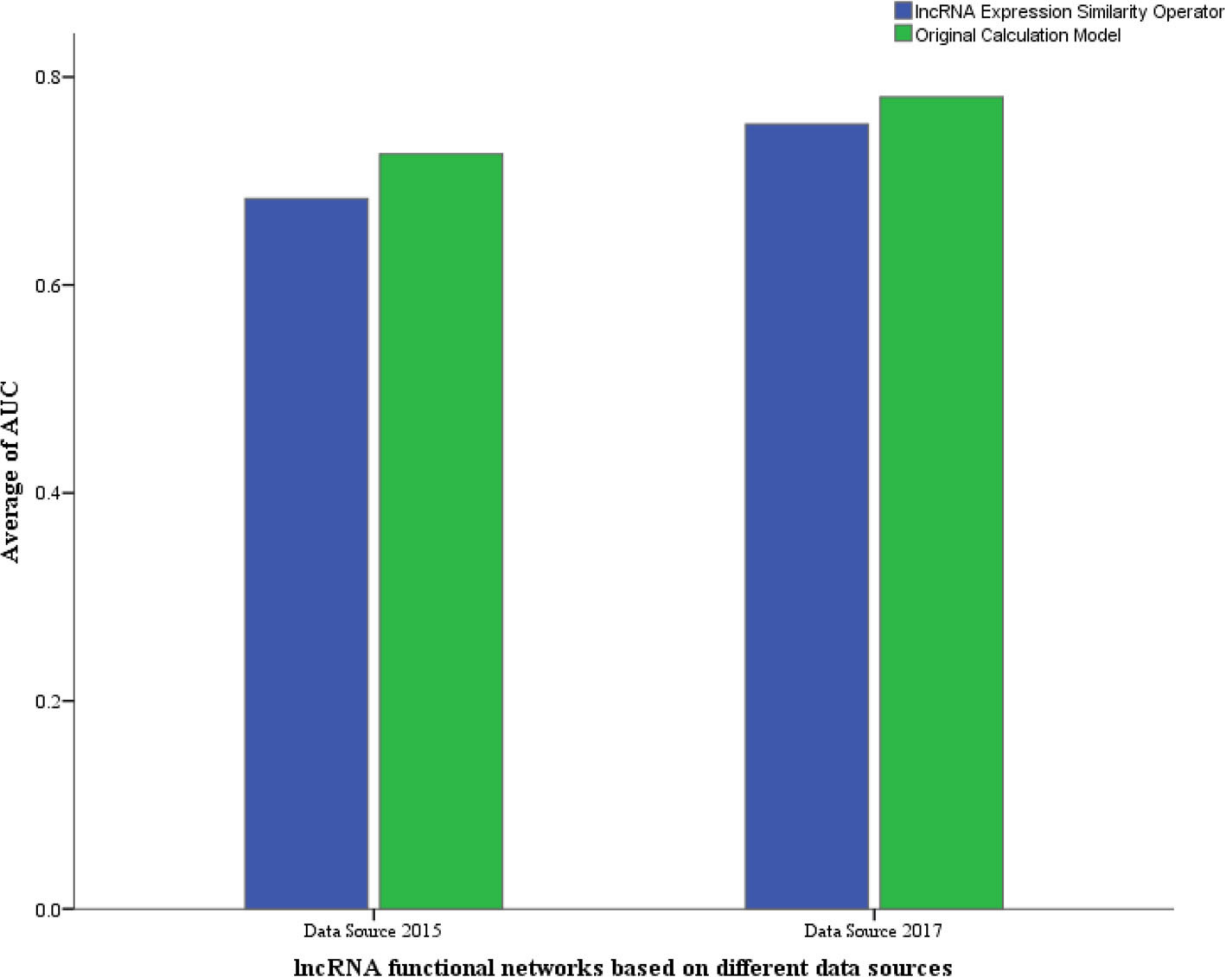
Average AUC of lncRNA functional networks built by different calculation models

Subsequently, we built two lncRNA functional networks based on data sources from the 2015 and 2017 versions with the lncRNA expression similarity operator introduced. LncRNADisease released in June 2018 was taken as the test set. The lncRNA functional network based on LncRNADisease released in 2015 had an AUC value of 68.3%, while the AUC of the network based on the 2017 version achieved 75.46%. As shown in Fig. 7, the original calculation model had a better performance than one with the lncRNA expression similarity operator introduced, regardless of whether the data source was extracted from the 2015 or 2017 version of LncRNADisease. We found that the number of mappings between RNAcentral and NONCODE was insufficient. This may have an impact on the performance because sufficient and reliable data can make a contribution to predicting potential disease-related lncRNAs, while a small amount of data may have a negative impact. Hence, this is the reason why the lncRNA expression similarity operator was not introduced.

## Conclusions

In this article, a computational model for potential disease-related lncRNA identification was proposed based on multiple biological datasets. The results showed that LncDisAP was proven to be successful in predicting novel disease-related lncRNA signatures with an average AUC value of 78.08% and can be an effective solution to improve the quality of disease diagnostics and treatments. To further evaluate the performance of our computational model, we used several common cancers as case studies. We found some unknown lncRNAs that could be associated with these diseases through our network. In addition, we discussed the impact of different data sources and different test sets on the performance of the disease-related lncRNA functional network in predicting disease-lncRNA pairs.

## Supplementary information

**Additional file 1:** Statistics of 54 diseases for evaluating disease-related lncRNA functional network.

## Data Availability

All the datasets used in this paper could be downloaded from websites.

## References

[1] Mercer TR, Dinger ME, Mattick JS (2009). Long non-coding RNAs: insights into functions. Nat Rev Genet.

[2] Iyer MK, Niknafs YS, Rohit M, Udit S, Anirban S, Yasuyuki H, Barrette TR, Prensner JR, Evans JR, Shuang Z (2015). The landscape of long noncoding RNAs in the human transcriptome. Nat Genet.

[3] Brunner AL, Beck AH, Edris B, Sweeney RT, Zhu SX, Rui L, Montgomery K, Varma S, Gilks T, Guo X (2012). Transcriptional profiling of long non-coding RNAs and novel transcribed regions across a diverse panel of archived human cancers. Genome Biol.

[4] Quan N, Carninci P (2015). Expression specificity of disease-associated lncRNAs: toward personalized medicine. Curr Top Microbiol Immunol.

[5] Harries LW (2012). Long non-coding RNAs and human disease. Biochem Soc Trans.

[6] Beermann J, Piccoli MT, Viereck J, Thum T (2016). Non-coding RNAs in development and disease: background, mechanisms, and therapeutic approaches. Physiol Rev.

[7] Wang J, Samuels DC, Zhao S, Xiang Y, Zhao YY, Guo Y (2017). Current research on non-coding ribonucleic acid (RNA). Genes.

[8] Shi X, Sun M, Liu H, Yao Y, Song Y (2013). Long non-coding RNAs: a new frontier in the study of human diseases. Cancer Lett.

[9] Batista P, Chang H (2013). Long noncoding RNAs: cellular address codes in development and disease. Cell.

[10] Wang YQ, Bai W, Wang MJ, Yu T, Zhang W (2019). Long non-coding RNA brain cytoplasmic RNA 1 acts as an oncogene and regulates cell proliferation and metastasis in non-small cell lung Cancer. J Nanosci Nanotechnol.

[11] Wang J, Cao L, Wu J, Wang Q (2018). Long non-coding RNA SNHG1 regulates NOB1 expression by sponging miR-326 and promotes tumorigenesis in osteosarcoma. Int J Oncol.

[12] Ye J-J, Cheng Y-L, Deng J-J, Tao W-P, Wu L (2019). LncRNA LINC00460 promotes tumor growth of human lung adenocarcinoma by targeting miR-302c-5p/FOXA1 axis. Gene.

[13] Aksoy F, Aksoy S, Tunca B, Işik O, Ozturk E, Yilmazlar T, Yerci O, Egeli U, Cecener G: The clinical significance of lncRNA DANCR in upper rectal adenocarcinoma. In: Annals of Oncology*:* 2018.

[14] Hajjari M, Salavaty A (2015). HOTAIR: an oncogenic long non-coding RNA in different cancers. Cancer Biology & Medicine.

[15] Zhao M, Wang S, Li Q, Ji Q, Guo P, Liu X. MALAT1: a long non-coding RNA highly associated with human cancers (review). Oncol Lett. 2018. 10.3892/ol.2018.8613 PMC6006327 29928382

[16] Chen C, Feng Y, Wang X. LncRNA ZEB1-AS1 expression in cancer prognosis: review and meta-analysis. Clin Chim Acta. 2018. 10.1016/j.cca.2018.06.007 29885321

[17] Harrow J, Frankish A, Gonzalez JM, Tapanari E, Diekhans M, Kokocinski F, Aken BL, Barrell D, Zadissa A, Searle S (2012). GENCODE: the reference human genome annotation for the ENCODE project. Genome Res.

[18] Fang SS, Zhang LL, Guo JC, Niu YW, Wu Y, Li H, Zhao LH, Li XY, Teng XY, Sun XH: NONCODEV5: a comprehensive annotation database for long non-coding RNAs. *Nucleic Acids Research* 2017, 46(Database issue). 10.1093/nar/gkx1107 PMC5753287 29140524

[19] Volders PJ, Verheggen K, Menschaert G, Vandepoele K, Martens L, Vandesompele J, Mestdagh P (2015). An update on LNCipedia: a database for annotated human lncRNA sequences. Nucleic Acids Res.

[20] Cheng L, Shi H, Wang Z, Hu Y, Yang H, Zhou C, Sun J, Zhou M (2016). IntNetLncSim: an integrative network analysis method to infer human lncRNA functional similarity. Oncotarget.

[21] Chen X, Yan CC, Luo C, Ji W, Zhang Y, Dai Q (2015). Constructing lncRNA functional similarity network based on lncRNA-disease associations and disease semantic similarity. Sci Rep.

[22] Gu C, Liao B, Li X, Cai L, Li Z, Li K, Yang J (2017). Global network random walk for predicting potential human lncRNA-disease associations. Sci Rep.

[23] Liang C, Li J, Peng J, Peng J, Wang Y (2014). SemFunSim: a new method for measuring disease similarity by integrating semantic and gene functional association. PLoS One.

[24] Szklarczyk D, Morris JH, Cook H, Kuhn M, Wyder S, Simonovic M, Santos A, Doncheva NT, Roth A, Bork P (2017). The STRING database in 2017: quality-controlled protein–protein association networks, made broadly accessible. Nucleic Acids Res.

[25] Li Jun-Hao, Liu Shun, Zhou Hui, Qu Liang-Hu, Yang Jian-Hua (2013). starBase v2.0: decoding miRNA-ceRNA, miRNA-ncRNA and protein–RNA interaction networks from large-scale CLIP-Seq data. Nucleic Acids Research.

[26] Kibbe WA, Arze C, Felix V, Mitraka E, Bolton E, Fu G, Mungall CJ, Binder JX, Malone J, Vasant D (2015). Disease ontology 2015 update: an expanded and updated database of human diseases for linking biomedical knowledge through disease data. Nucleic Acids Res.

[27] Davis A. P., Wiegers T. C., Rosenstein M. C., Mattingly C. J. (2012). MEDIC: a practical disease vocabulary used at the Comparative Toxicogenomics Database. Database.

[28] Chen G, Wang Z, Wang D, Qiu C, Liu M, Chen X, Zhang Q, Yan G, Cui Q (2013). LncRNADisease: a database for long-non-coding RNA-associated diseases. Nucleic Acids Res.

[29] Library WP (2009). Human protein reference database.

[30] Davis AP, Grondin CJ, Johnson RJ, Sciaky D, King BL, McMorran R, Wiegers J, Wiegers TC, Mattingly CJ (2017). The comparative Toxicogenomics database: update 2017. Nucleic Acids Res.

[31] Library WE (2000). Medical subject headings (MeSH). Bull Med Libr Assoc.

[32] Consortium TR (2017). RNAcentral: a comprehensive database of non-coding RNA sequences. Nucleic Acids Res.

[33] Bao Z, Yang Z, Huang Z, Zhou Y, Cui Q, Dong D: LncRNADisease 2.0: an updated database of long non-coding RNA-associated diseases. *Nucleic acids research* 2018. 10.1093/nar/gky905 PMC6324086 30285109

[34] Wang Y, Juan L, Chu Y, Wang R, Zang T, Wang Y: FNSemSim: an improved disease similarity method based on network fusion. In: IEEE International Conference on Bioinformatics and Biomedicine*:* 2017. 630–633.

[35] Tong H, Faloutsos C, Pan J-Y: **Fast random walk with restart and its applications**. In: *Icdm 2006: Sixth International Conference on Data Mining, Proceedings.* Edited by Clifton CW, Zhong N, Liu JM, Wah BW, Wu XD; 2006: 613−+.

[36] Liz J, Esteller M (2015). lncRNAs and microRNAs with a role in cancer development. Biochim Biophys Acta.

[37] Zhang T, Zhou J, Zhang W, Lin J, Ma J, Wen X, Yuan Q, Li X, Xu Z, Qian J (2018). H19 overexpression promotes leukemogenesis and predicts unfavorable prognosis in acute myeloid leukemia. Clin Epigenetics.

[38] Zhao TF, Jia HZ, Zhang ZZ, Zhao XS, Zou YF, Zhang W, Wan J, Chen XF (2017). LncRNA H19 regulates ID2 expression through competitive binding to hsa-miR-19a/b in acute myelocytic leukemia. Mol Med Rep.

[39] Tang J, Li Y, Sang Y, Yu B, Lv D, Zhang W, Feng H. LncRNA PVT1 regulates triple-negative breast cancer through KLF5/beta-catenin signaling. Oncogene. 2018. 10.1038/s41388-018-0310-4 29760406

[40] Aboelsoud MM, Chaiteerakij R, Giama NH, Moser CD, Baichoo E, Mettler TA, Juran BD, Harmsen WS, Therneau TM, Lazaridis K (2014). Genetic Polymorphisms in the COX2 and Wrap53 Genes Are Associated With Risk but Not Survival of Cholangiocarcinoma. Gastroenterology.

[41] Shi X, Zhang H, Wang M, Xu X, Zhao Y, He R, Zhang M, Zhou M, Li X, Peng F (2017). LncRNA AFAP1-AS1 promotes growth and metastasis of cholangiocarcinoma cells. Oncotarget.

[42] Lu X, Zhou C, Li R, Deng Y, Zhao L, Zhai W (2017). Long noncoding RNA AFAP1-AS1 promoted tumor growth and invasion in Cholangiocarcinoma. Cell Physiol Biochem.

[43] Zhu H, Zheng T, Yu J, Zhou L, Wang L (2018). LncRNA XIST accelerates cervical cancer progression via upregulating Fus through competitively binding with miR-200a. Biomedicine & pharmacotherapy = Biomedecine & pharmacotherapie.

